# A new direction for differentiating animal activity based on measuring angular velocity about the yaw axis

**DOI:** 10.1002/ece3.6515

**Published:** 2020-07-06

**Authors:** Richard M. Gunner, Rory P. Wilson, Mark D. Holton, Rebecca Scott, Phil Hopkins, Carlos M. Duarte

**Affiliations:** ^1^ Swansea Lab for Animal Movement, Biosciences College of Science Swansea University Swansea UK; ^2^ Red Sea Research Centre King Abdullah University of Science and Technology Thuwal Saudi Arabia; ^3^ Future Ocean Cluster of Excellence GEOMAR Helmholtz Centre for Ocean Research Kiel Germany; ^4^ Natural Environmental Research Council, Polaris House Swindon UK

**Keywords:** accelerometer, angular velocity, animal behavior, animal heading, magnetometer, sea turtle

## Abstract

1. The use of animal‐attached data loggers to quantify animal movement has increased in popularity and application in recent years. High‐resolution tri‐axial acceleration and magnetometry measurements have been fundamental in elucidating fine‐scale animal movements, providing information on posture, traveling speed, energy expenditure, and associated behavioral patterns. Heading is a key variable obtained from the tandem use of magnetometers and accelerometers, although few field investigations have explored fine‐scale changes in heading to elucidate differences in animal activity (beyond the notable exceptions of dead‐reckoning).
2. This paper provides an overview of the value and use of animal heading and a prime derivative, angular velocity about the yaw axis, as an important element for assessing activity extent with potential to allude to behaviors, using “free‐ranging” Loggerhead turtles (*Caretta caretta*) as a model species.
3. We also demonstrate the value of yaw rotation for assessing activity extent, which varies over the time scales considered and show that various scales of body rotation, particularly rate of change of yaw, can help resolve differences between fine‐scale behavior‐specific movements. For example, oscillating yaw movements about a central point of the body's arc implies bouts of foraging, while unusual circling behavior, indicative of conspecific interactions, could be identified from complete revolutions of the longitudinal axis.
4. We believe this approach should help identification of behaviors and “space‐state” approaches to enhance our interpretation of behavior‐based movements, particularly in scenarios where acceleration metrics have limited value, such as for slow‐moving animals.

## Graphical abstract

We consider the extent to which angular velocity (particularly yaw rotation) can inform biologists of animal activity and behaviour. We advocate this as particularly powerful in scenarios where standard acceleration metrics have limited value, such as in slow‐moving animal taxa.

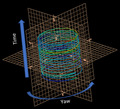

## 1 BACKGROUND

Animals can enhance their fitness by responding behaviorally according to their physiological state and environmental circumstance (Nathan et al., 2008; Shepard et al., 2013; Wilmers et al., 2015). At its inception, animal behavior was defined by direct observation of body posture and limb movement (Brown, Kays, Wikelski, Wilson, & Klimley, 2013; Patterson, Thomas, Wilcox, Ovaskainen, & Matthiopoulos, 2008). More recently, however, researchers have realized that animal‐attached tags can help define behavior using tri‐axial acceleration data, which resolve both animal posture and the dynamism of movement (Shepard, Wilson, Quintana et al., 2008; Wilson, Wikelski, Wilson, & Cooke, 2015; Yoda et al., 2001; Zimmer, Ropert‐Coudert, Kato, Ancel, & Chiaradia, 2011). Indeed, animal‐attached accelerometers are now widespread and common for both short‐term and long‐term continuous monitoring of the behavior of wild animals (Brown et al., 2013; Wilmers et al., 2015). Methods used to identify animal behaviors include random forests (e.g., Fehlmann et al., 2017), neural networks (e.g., Samarasinghe, 2016), and support vector machines (e.g., Martiskainen et al., 2009) and generally tease out accelerometer‐derived data streams using both raw accelerometer data and derivatives, such as VeDBA (e.g., Benoit et al., 2020, Patterson et al. 2019), to find some combination of values that characterize specific behaviors.

Not all behaviors can be accurately resolved from accelerometers though (Williams et al., 2017) because slow‐moving animals, such as some reptiles (Walker & Westneat, 2000; Wyneken, 1996), may produce negligible changes in the recorded dynamic acceleration (for definition, see Wilson et al., 2020). Similar issues arise during bouts of gliding behavior, as exhibited by many marine and aerial species that can maintain relatively constant velocity for long periods of time (Eckert, 2002; Williams, Shepard, Duriez, & Lambertucci, 2015). In addition, external forces acting on the animal, such as current vectors in air or water, can decrease the signal‐to‐noise ratio of measured acceleration, confounding the interpretation of data (cf. Halsey, Shepard, & Wilson, 2011; Robert‐Coudert & Wilson, 2004). Animal posture derived from acceleration also becomes problematic when animals are subject to high centripetal acceleration (e.g., when a bird banks sharply (Clark, 2009; Williams et al., 2015)) or when substrate beneath the study animal varies in declination (Wilson, Holton, et al., 2018; Wilson, Neate, et al., 2018). Finally, a critical limitation of accelerometers is that they cannot resolve rotation about the yaw axis (typically referred to as; “heading”) even though movement in this axis is at least as important as rotations in the other two axes: pitch and roll (Kano, Walker, Sasaki, & Biro, 2018; Noda, Okuyama, Koizumi, Arai, & Kobayashi, 2012).

Both Chakravarty, Maalberg, Cozzi, Ozgul, and Aminian (2019) and Williams et al. (2017) have suggested that tri‐axial magnetometers could be used as behavioral identification sensors in manner analogous to tri‐axial accelerometers. Certainly, magnetometers have appreciable advantages over accelerometers because they are unaffected by both gravitational and dynamic components of acceleration (López, de Soto, Miller, & Johnson, 2016; Noda, Kawabata, Arai, Mitamura, & Watanabe, 2014) and, unlike gyroscopes, are not subject to drift over larger time intervals (Fong, Ong, & Nee, 2008), also requiring less electric current and can be usefully sampled at lower frequencies (Caruso, 2000). We note though that gyroscopes have been used in a suite of biologging studies to determine angular velocity (cf. Noda et al., 2012, Noda, Kawabata, Arai, Mitamura, & Watanabe, 2013, Gerencsér, Vásárhelyi, Nagy, Vicsek, & Miklósi, 2013, Kawabata et al., 2014). Tri‐axial magnetometers react to variations in magnetic field orientation and intensity in all three spatial dimensions (López et al., 2016; Wilson et al., 2007). Orientation about the yaw axis of an animal‐attached tag is accessed by consideration of the animal's pitch and roll (from accelerometers) in relation to the output of the tri‐axial magnetic field sensors and is described in detail by Bidder et al. (2015). This approach normally allows the animal heading to be defined to within about 1–2° (Painter et al., 2016).

Importantly, derivation of heading, and associated metrics (see later), should provide additional important measurements to define activity and with which behaviors may be identified using techniques such as machine learning (Wang et al., 2015) and time‐based decision trees (Rutkowski, Jaworski, & Duda, 2020) as well as being important in dead‐reckoning studies (Laplanche, Marques, & Thomas, 2015). Crucially, magnetometers reveal patterns in movement at various scales of rotation along the yaw axis that are not always evident in acceleration data (López, Miller, de Soto, & Johnson, 2015; López et al., 2016; Williams et al., 2015, 2017), which should prove particularly informative for behaviors that produce no definitive acceleration‐based pattern and for animals that generate negligible dynamic acceleration signal.

This paper examines the value and use of animal heading and a prime derivative, angular velocity about the yaw axis (hereafter termed AVeY), as an element for quantifying activity and ultimately aspiring to help differentiate animal behavior using the Loggerhead turtle (*Caretta caretta*) as a model species. Firstly, we propose several metrics derived from AVeY and discuss how various temporal scales over which AVeY (and derivatives) is calculated, in some cases, can be a sensitive indicator of to some types of movement beyond those which acceleration alone can detail. Secondly, we outline the wider implications of using such AVeY metrics to aid in examining fine‐scale behaviors. Lastly, we assess the relationship between AVeY and the commonly used DBA‐based proxy for energy expenditure; vectorial dynamic body acceleration (VeDBA) (cf. Miwa et al., 2015) to see how the two metrics scale. The general aims of this research are to examine the potential importance in AVeY for enhancing our understanding of animal movement and to provide a framework for deriving and utilizing indices of AVeY for researchers investigating proximate and ultimate aspects of animal behavior using the technology outlined above.

## 2 MATERIALS AND METHODS

### 2.1 Subjects, study area, and tagging

The attachment of data loggers was carried out between July and August 2014 on five mature female loggerhead turtles, intercepted, and tagged immediately post egg‐laying on the Southern beaches of Boa Vista, Cape Verde Islands (15°58′22″ N, 22°47′56″ W). Daily diary (DD) logging units (Wilson, Shepard, & Liebsch, 2008) were used in this study. Acceleration measurements were logged as acceleration with respect to gravity (1 g = 9.81 m/s) from each of the three orthogonally mounted sensor axes (measuring within the range of ± 16 g). Orthogonal magnetometry measurements were recorded in Gauss (G) (magnetic intensity recorded within the range of ±0.88 G at 0.73 mG/LSB resolution). Both accelerometery and magnetometry data were logged at 40 Hz. The DDs also recorded temperature (°C) and pressure (mbar). Data were stored on a 2 GB removable micro‐SD card. The DDs were enclosed in an oval‐cylinder water‐tight nylon casing and powered with one A‐cell battery. Tags were attached onto the second dorsal scute of the turtle carapace, using a two‐component epoxy resin glue, to ensure tag housings (and thus sensors) were placed as close as possible to the horizontal. Tags were positioned so that the *x*, *y*, and *z* axes of both the accelerometer and magnetometer sensors were aligned to the anterior‐posterior (surge), medio‐lateral (sway) and dorsal‐ventral (heave) axes of the animal, respectively. Tags were retrieved after a single internesting interval (approximately 2 weeks after initial deployment).

### 2.2 Derivation of VeDBA

Vectorial dynamic body acceleration (VeDBA) is a single integrated measure of the vector sum of dynamic acceleration from the three spatial dimensions during a given inertial frame (Qasem et al., 2012). To calculate VeDBA, a 2‐s running mean was applied to each of the acceleration axes in order to approximate the static acceleration (McClune et al., 2014; Shepard, Wilson, Halsey, et al., 2009). The static acceleration from each axis was subtracted from the raw acceleration values from each axis to derive the dynamic acceleration (Gleiss, Wilson, & Shepard, 2011, Shepard et al., 2013). VeDBA was determined by taking the vectorial sum of the dynamic acceleration using:

(1) $$\text{VeDBA}=\sqrt{\left(x^{2}+y^{2}+z^{2}\right)},$$

where *x*, *y*, and *z* are the derived dynamic acceleration values from each axis.

### 2.3 Derivation of angular velocity

The static component of acceleration (due to gravity, which amounts to 1 g) is used in the computation of rotation about the three axes and is typically achieved by passing a moving average of a given window size *w* (2 s used in this study) through a given sample (***S_i_***) of each orthogonal channel's acceleration *via:*

(2) $$S_{i}=\frac{1}{w}\sum_{j=i‐\frac{w}{2}}^{i+\frac{w}{2}}S_{j}$$

Pitch and roll are computed *via*:

$\text{Pitch}\left(\beta \right)=\left(a\tan2\left(S_{x},\sqrt{S_{y}∙S_{y}+S_{z}∙S_{z}}\right)\right)∙\frac{180}{\pi }$

(3) $$\text{Roll}\left(Y\right)=\left(a\tan2\left(S_{y},\sqrt{S_{x}∙S_{x}+S_{z}∙S_{z}}\right)\right)∙\frac{180}{\pi }$$

where *S_x_*_,_ *_y_*_,_ *_z_* refer to the static components of acceleration from the *x*, *y*, and *z* channels of the accelerometer, respectively (Bidder et al., 2015).

For review of all stages and mathematical components involved in the derivation of compass heading (*H*) (yaw), see Bidder et al. (2015) and Walker et al. (2015). In brief however, the magnetometer is typically calibrated by rotating the tag so that all orientations of roll, pitch, and yaw are covered (Williams et al., 2017). In the absence of distortions to the local magnetic field, the normalized data from each axis (of this calibration period) form a sphere when plotted on a tri‐axial magnetic field intensity scatter‐plot (the “m‐sphere”; Williams et al., 2017). This process essentially provides a reference frame of the vectorial sum of magnetometry data across all three spatial dimensions and enables subsequent compensation for “hard” and “soft iron” errors. Soft iron deposits distort the magnetic field around the device, causing the sphere to take on an ovoid/ellipsoid form (Gebre‐Egziabher, Elkaim, David Powell, & Parkinson, 2006; Ozyagcilar, 2012). Hard iron deposits introduce a constant bias, shifting the position of the magnetic field and thus position of the sphere away from its true origin (Gebre‐Egziabher et al., 2006; Ozyagcilar, 2012). An ellipsoid‐fitting algorithm and correction factor are used to correct such distortions and re‐form uniform spherical fields about the true origin (Bidder et al., 2015; Walker et al., 2015). Angular rotations about the pitch and roll axes derived from the static component of acceleration are subsequently used in the tilt correction procedure on each orthogonal magnetometer channel. Compass data are first normalized, and then, each orthogonal channel is rotated according to the pitch and roll. This ensures magnetometer outputs are compensated, according to the inclination and declination angles caused by postural offsets, with outputs corrected to give a horizontal co‐ordinate frame (Bidder et al., 2015). Finally, compass heading (*H*) with respect to magnetic North is achieved *via*:

(4) $$H=\mathrm{mod}\left(360+\left(a\tan2\left(‐m_{y},m_{x}\right)∙\frac{180}{\pi }\right),360\right),$$

where *m_x_*_,_ *_y_* refer to the normalized, ellipse fitted and co‐ordinate frame‐adjusted *x* and *y* channels of the magnetometer, respectively (Bidder et al., 2015) and mod refers to the modulo operator. The heading output uses a scale of 0°–360°, with Magnetic North equating to a heading of 0° or 360°, South to 180°, and thus East and West to 90° and 270°, respectively.

### 2.4 Data analysis

All data analysis was performed in the custom designed software; Daily Diary Multi Trace (DDMT) (http://www.wildbytetechnologies.com), RStudio (open source statistical programming software, http://www.R‐project.org), Origin pro 2016 (OriginLab Corporation, http://www.originlab.com), and Microsoft Excel 2016. Only flat U‐shaped dives (type 1a) greater than or equal to 3 meters in depth (cf. Houghton, Broderick, Godley, Metcalfe, & Hays, 2002) were used in the analysis to illustrate points being made.

DDMT enables 2D visualizations of infra‐second variation per sensor axis output (visualized as waveforms over time) and includes built‐in features for quantifying and exporting channel subsets, calculating channel differentials and derivatives (including VeDBA), altering channel smoothing windows, producing multi‐dimensional plots and an interface for searching for behaviors, and bookmarking them using a Boolean time‐based approach (Wilson, Holton, et al., 2018; Wilson, Neate, et al., 2018). DDMT also provides the platform to correct for iron distortions and tilt offsets from calibration data, before computing compass heading. All three indices of derived body rotation (pitch, roll, and yaw) were presmoothed using a rolling window of 2 s in order to reduce small deviations due to noise (i.e., small deviations manifest by the flipper beat cycle). Pitch and roll values ranged from −90° to +90°. The arithmetic mean of the yaw axis is problematic due to the periodic nature of circular data (range from 0° to 360°; both of which define the exact same point on the circumference of the unit circle—magnetic North (cf. Pewsey, Neuhäuser, & Ruxton, 2013). Therefore, units were converted from degrees to Cartesian coordinates, before calculating the arithmetic mean of the individual angles from sample trigonometric moments and finally restoring resultant units back to degrees *via:*

(5) $${\bar{\theta }}_{p}=a\tan2\left(\frac{1}{n}\sum_{j=i}^{n}\sin(H_{j}∙\frac{\pi }{180}),\frac{1}{n}\sum_{j=i}^{n}\cos(H_{j}∙\frac{\pi }{180})\right)$$

(6) $$\bar{H}=\mathrm{mod}\left(360+\left({\bar{\theta }}_{p}∙\frac{180}{\pi }\right),360\right).$$

Each dataset was subsequently subsampled to 1 Hz (selected values at *i* = 40) to make the data more manageable and because turtles are generally slow‐moving anyway (as manifest by extremely low VeDBA values, typically lower than 0.08 g). Differentials were then calculated from the smoothed values of each axis, using a stepping range of 1 s (^o^/s) and termed: AVeP, AVeR, and AVeY (angular velocity about the pitch, roll, and yaw axis, respectively). AVeY was also calculated over two larger temporal scales: 5 s (AVeY (^o^/5_s_)) and 10 s (AVeY (^o^/10_s_)). Since compass heading is circular, with no true zero and any designation of low or high values being arbitrary, a logical expression was implemented on the derivative AVeY to ensure rate of change never exceeded 180 °/s, whereby 360 was added to AVeY values less than −180 and 360 was subtracted from AVeY values greater than 180. This makes biological sense as long as the time interval over which the angular velocity is calculated is restricted because it is far more likely that an animal that caused the compass heading to change from 10° to 350°, turned 20° anticlockwise, rather than 330° clockwise. Careful consideration of the animal in question must be made when choosing the sampling period of AVeY, to avoid inaccurate rates of change. Ideally, this should be less than the time it takes the animal to rotate through half a revolution.

The maximum angle that could be yielded from any given axis per second was therefore 180°. A metric termed absolute angular velocity (AAV) was derived from the integration of each of the rotational axes' absolute instantaneous angular velocity measurement:

(7) $$\text{AAV}=\sqrt{\left({\text{AVeP}}^{2}+{\text{AVeR}}^{2}+{\text{AVeY}}^{2}\right)}$$

Conditional running cumulative sum functions in R were implemented on AVeY (^o^/s) to document each time a turn (or multitude of turns) in either direction, totaled/surpassed; 20°, 45°, 90°, and 180^o^ with respect to a particular starting orientation, resetting each time the condition (angle of specified yaw rotation to exceed) was met. The degree of yaw rotation in either direction with respect to the starting orientation and the specified turn angle with which it was equated was expressed as a percentage coverage over time (0 to ±100%; “+” representing clockwise, “−” representing anticlockwise). A final new metric, termed “cumulative heading” (CuHe), also implemented on AVeY (^o^/s), assessed the percentage coverage (0%–100%) about the yaw axis from the culmination of angular rotations in both directions, resetting each time the animal had (at least once), rotated through all 360°.

A linear mixed model (LMM) was performed utilizing the “lmer” function in R, from the “lme4” package (Bates, Mächler, Bolker, & Walker, 2014), to determine the relationship between mean values of AVeY (^o^/s) and VeDBA per dive. Turtle ID was included as a random factor in the model (Kuznetsova, Brockhoff, & Christensen, 2017). Three outliers were removed from the model following diagnostic plots of residuals. A Random slope model (including random intercepts) was also constructed to examine whether there was a significant difference between the slope coefficients, as given from the interaction of AVeY by turtle ID. The model simplification method was employed using likelihood ratio tests. Spearman rank correlation coefficients (*r*_s_) were derived to determine the association of ranks between various metrics of body rotation (detailed in Table 1) and VeDBA.

**TABLE 1:** Description of the metrics derived from accelerometer and magnetometer outputs

Metric	Abbreviation	Description	Smoothing	Unit
Angular velocity about the yaw axis	AVeY	AVeY (°/s^−1^) = *x*_i + 1_ − *x_i_*, AVeY (°/5s^−1^) = *x*_i + 5_ − *x_i_*, AVeY (°/10s^−1^) = *x_i_*_ + 10_ − *x_i_*, where *x* is the *i*th value of heading	Yaw presmoothed by 2 s (circular mean) prior to calculating differentials	°/s °/5s °/10s
Angular velocity about the pitch axis	AVeP	AVeP = *x_i_*_ + 1_ – *x_i_*, where *x* is the *i*th value of pitch	Pitch presmoothed by 2 s prior to calculating differential	°/s
Angular velocity about the roll axis	AVeR	AVeR = *x_i_*_+1_ − *x_i_*, where *x* is the *i*th value of roll	Roll presmoothed by 2 s prior to calculating differential	°/s
Absolute angular velocity	AAV	$\text{AAV}=\sqrt{\left({\text{AVY}}^{2}+{\text{AVP}}^{2}+{\text{AVR}}^{2}\right)}$	N/A	°/s
Vectorial dynamic body acceleration	VeDBA	$\text{VeDBA}=\sqrt{\left(x^{2}+y^{2}+z^{2}\right)}$, where *x*, *y,* and *z* are the derived dynamic acceleration values from each axis	2 s	*g*

## 3 RESULTS

**FIGURE 1:**
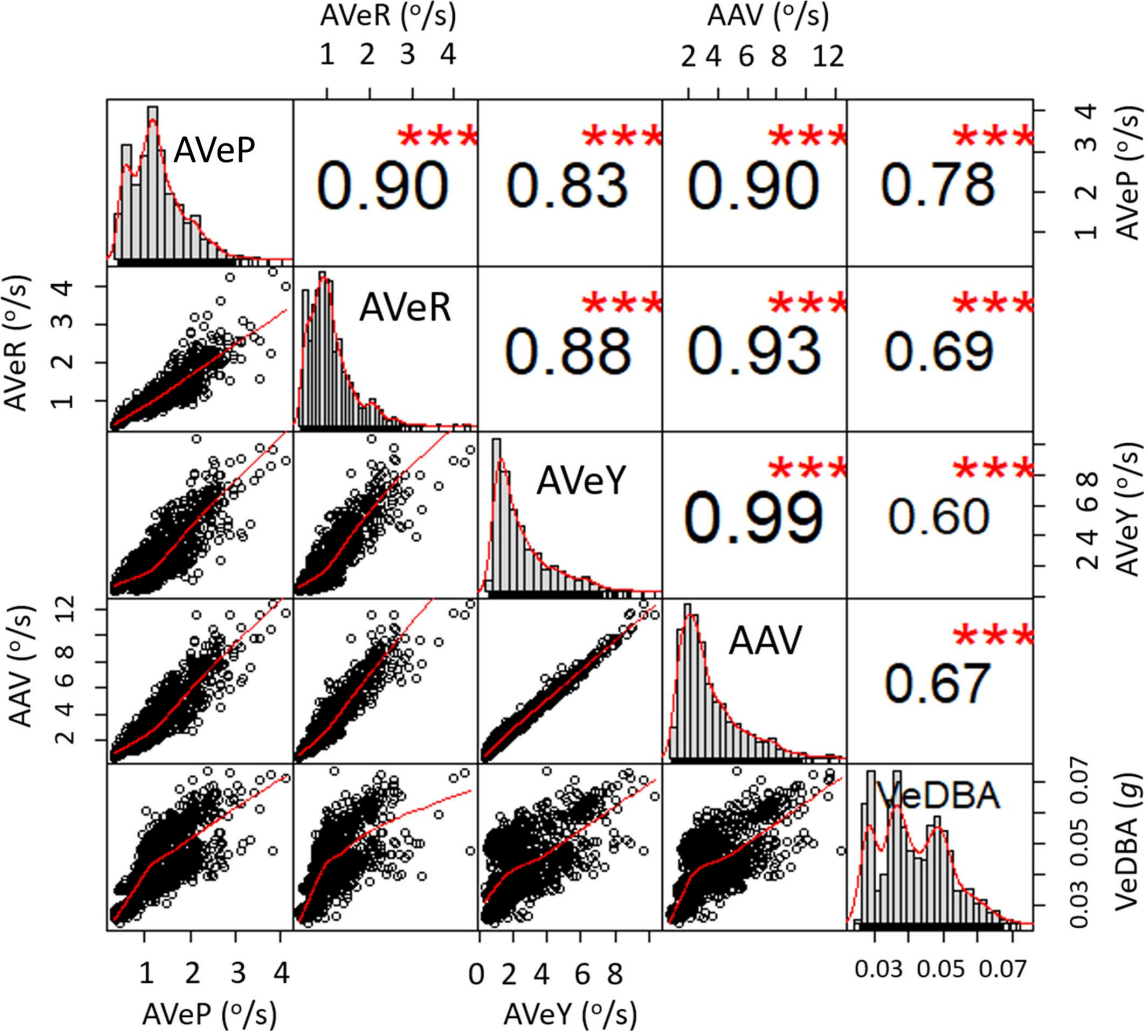
Association between the various metrics of body rotation (outlined in Table 1) and VeDBA, with strength of correlation coefficients (Spearman rank) and significance level detailed (*** = 0.01 significance level). Each data point represents the mean value per flat U‐dive (bottom phase duration)

There were strong positive significant correlations between the three differentials of body rotation, with higher angular velocities of body orientation being apparent across all three axes of rotation (pitch, roll, and yaw) (Figure 1). Of the three rotational axes though, AVeY had the strongest correlation with AAV, signifying that yaw was the predominant axis of rotation during activity (for this study species at least).

**FIGURE 2:**
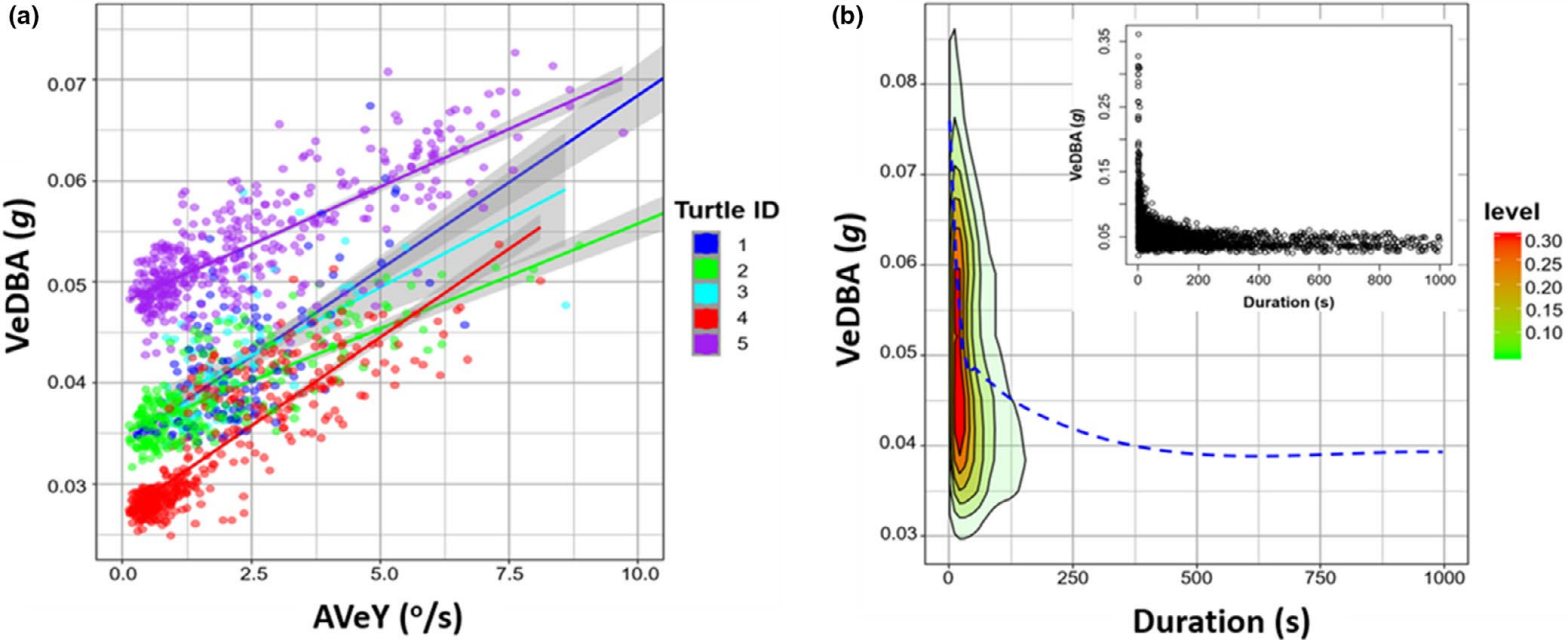
Association between yaw rotation and VeDBA, each data point represents the mean value per flat U‐dive (bottom phase duration). Relationship between AVeY (^o^/s) and VeDBA with implemented linear regressions and 95% confidence intervals (gray shading around each regression) (a). Data points and regression lines colored according to turtle ID. Contour plot showing the kernel density level of VeDBA as a function of the time it took to complete 90° turns, with “loess smooth” line (blue dashed) fitted (inset shows the raw *x*–*y* data) (b)

Model simplification and AIC values detailed the model incorporating both random slopes and random intercepts best improved goodness of fit; AVeY significantly affected VeDBA (LMM: *χ* ^2^(1) = 15.82, *p* < .01), with every 1 unit increase in AVeY increasing VeDBA by approximately 0.029 g (est. = 0.0029 ± 0.00027 (*SE*), *t* = 4.92, 95% CI[0.0022, 0.0035], *p *< .001) (Figure 2a). Incorporating random effects greatly improved the variance explained (marginal *R* ^2^ = .33; conditional *R* ^2^ = .84). In addition, the interclass correlation (ICC) was high at 0.79, indicating a high account of correlation among observations within groups. Typically, mean values of VeDBA were higher (and encompassed greater range) during faster turn rates (Figure 2b).

**FIGURE 3:**
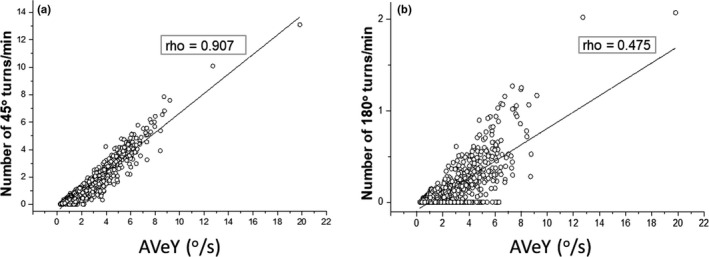
Correlation between values of AVeY (^o^/s) and the corresponding number of 45° turns (a) and 180° turns (b) per minute. Each data point represents the mean value per flat U‐dive (bottom phase duration)

Generally, greater values of AVeY corresponded in a linear fashion with the number of (specified) turn angles. This was particularly evident for 45° turns (Figure 3a). However, the relationship became weaker as the extent of the turn increased (Figure 3b), indicating that as the angle of a turn increased the chances of the animal turning back also increased.

**FIGURE 4:**
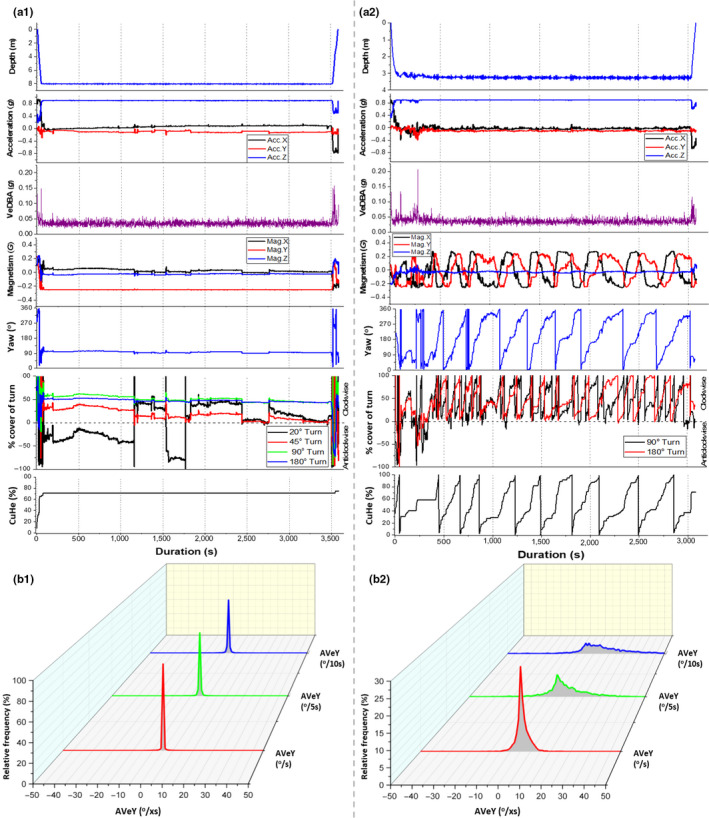
Two flat U‐dives similar in duration and differing in activity extent. Stacked line graphs detailing differences between acceleration‐ and magnetism‐based metrics plotted against dive duration. From top to bottom; depth profile, dynamic acceleration and derived VeDBA, magnetism and derived yaw, percentage coverage of various turn extents and CuHe (a). Frequency distribution of AVeY of both dives, calculated at three temporal scales (b)

We recognized that the bottom phase of turtle dives showed appreciable variability in recorded parameters, but that they could be broadly divided into apparently “active” and “inactive” (or “quiescent”) according to changes in yaw (cf. Figure S1). Both types involved little or no change in depth but in the quiescent dives, there were no definitive changes in either the acceleration or geomagnetism traces throughout the duration of the bottom phase, with yaw and VeDBA values also showing marked consistency (Figure 4a_1_). In such cases, CuHe remained static, showing that no “new” orientation was adopted following the initial descent (with only three 20° turn angles being surpassed during this period for the example in Figure 4a_1_). Frequency distributions were also similar between the three scales of AVeY, indicating changes in directionality were monotonous over time (Figure 4b_1_).

Conversely, “active” dives showed greater fluctuations in the geomagnetism traces and associated values of yaw, even though the depth, acceleration and VeDBA values showed similar patterns to apparently quiescent bottom phases of dives (cf. Figure 4a_2_). Full rotations about the yaw axis as depicted by CuHe were completed at a relatively consistent rate, as detailed by the percentage coverage of 90° and 180° turns and were nearly exclusively carried out in the clockwise direction, depicting a type of “circling” behavior (cf. Figure S2).

There was greater variation between the frequency distributions of the various rates of AVeY (Figure 4b_2_), all of which portrayed a greater spread and skewness compared to the quiescent dives (Figure 4b_1_), signifying a greater variability of turn rates, primarily carried out in one direction.

**FIGURE 5:**
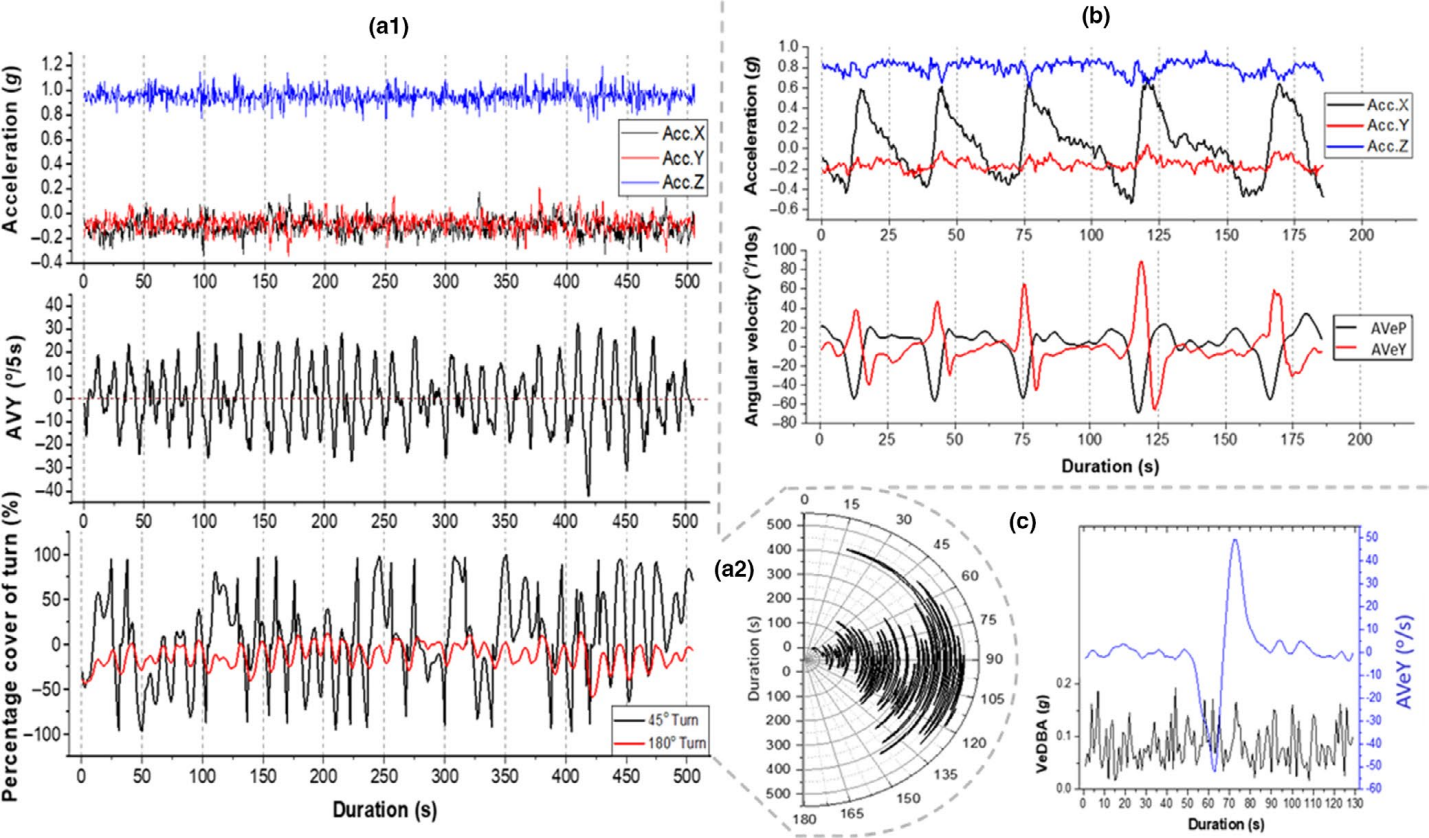
Various examples of activity‐specific patterns as resolved by examination of body rotations at various temporal scales and smoothing windows. Stacked line graphs showing the variation in acceleration, AVeY (^o^/5s) (smoothing window; 3 s) and percentage coverage of 45° and 180° turns over an eight‐minute period (a_1_). Polar theta chart detailing the changes in heading (from a_1_) over time (a_2_). Stacked line graphs showing the variation in acceleration, AVeP and AVeY (^o^/s) (smoothing window; 5 s) over a three‐minute period (b). Changes in VeDBA and AVeY (^o^/s) (smoothing window; 3 s) during a segment of acute turning behavior (c)

Calculating AVeY over different intervals and “postsmoothing” at various window lengths highlighted behavior‐specific patterns in signal minima and maxima of AVeY, such as clear alternating fluctuations in heading, even when there seemed no clear pattern observed from acceleration data (e.g., Figure 5a_1_). In the example shown in Figure 5a_1_, changes in heading resulted in 45° turns in both directions, yet 180° turns were never achieved, indicating an activity‐specific behavior with an overall directionality (Figure 5a_2_).

Conversely, patterns apparent in the acceleration data could be further investigated with respect to AVeY in order to enhance information of the behavior‐specific movement taking place. For example, Figure 5b Shows relatively consistent deviations recorded from the surge axis (Acc. X), manifest as in spikes of pitch, a pattern which coincided with acute anticlockwise turns although changes in orientation did not necessarily correspond with clear changes to dynamic acceleration estimates (Figure 5c).

**FIGURE 6:**
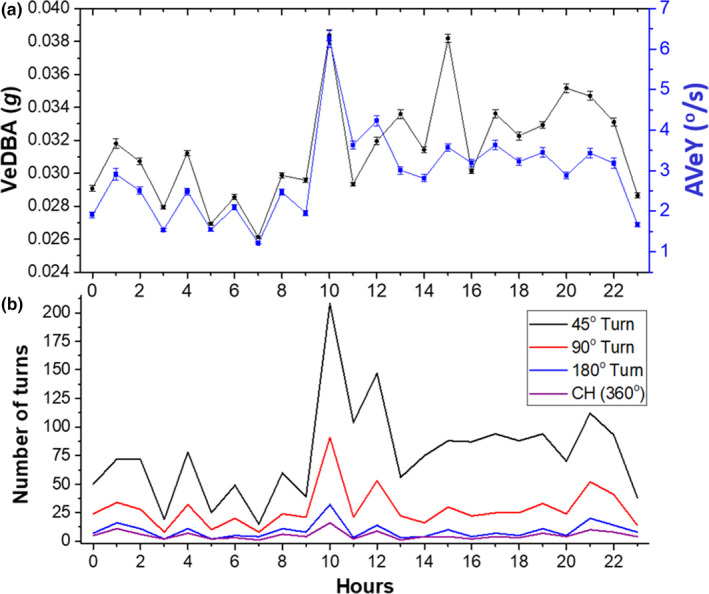
Time series plot of mean values of VeDBA and AVeY (^o^/s) (± *SE*) per hour over a 24‐hr period (a) and the corresponding number of 45°, 90°, and 180° turns and complete rotations about the yaw axis (CuHe) (b)

In the turtle data, the different yaw‐based metrics were often complementary, showing clear patterns in activity. For example, AVeY (and VeDBA) could show similar changes over a 24‐hr period (Figure 6a) even though higher turn rates did not necessarily correspond with turn extent (Figure 6b), indicating that different processes were at work over the sample period.

**FIGURE 7:**
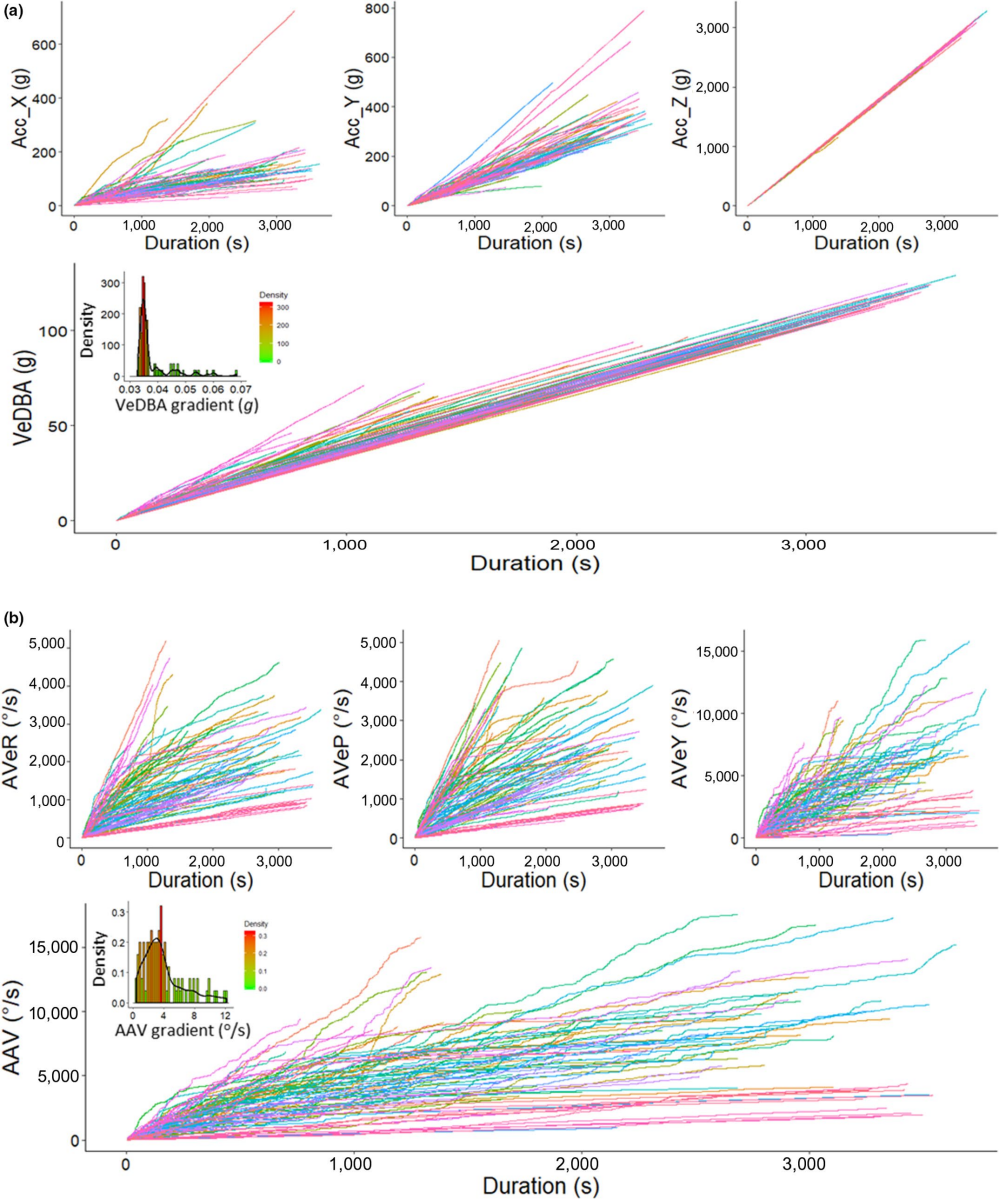
Cumulative frequency of VeDBA (g) (a) and AAV (^o^/s) (b) across the duration of 100 random U‐dives (bottom phase), including their precursor measurements; raw absolute values of acceleration (including both dynamic and static components) from the *x*‐ (surge), *y*‐ (sway), and *z*‐ (heave) channels (a) and absolute values of AVeR, AVeP, and AVeY (b). Frequency histogram distribution inlets given for cumulative VeDBA and AAV gradients per dive. Note the much larger spread and variability of patterns when cornering indices of angular rotation

Accumulating (absolute) values of angular rotation across duration of 100 random U‐dives demonstrated the variability both within and between dives (Figure 7). Such a spread of accumulated distributions was less pronounced for acceleration indices. Moreover, the frequency distribution of cumulated AAV gradients was less obviously monomodal than the equivalent VeDBA gradients. CuHe was another diverse yaw‐based metric, reflecting the degree and intensity of directionality involved in movement. Behaviors that were manifest by restricted, or no change in, direction, resulted in a lower accumulation of unique angles attained around the body's circumference, which was reflected in the small step ranges of CuHe and fewer resets to zero (when 100% of angles have been covered at least once). Conversely, other, ostensibly more active behaviors with little overall directionality (i.e., searching) resulted in frequent revolutions. Here, a distinct tri‐modal distribution of CuHe frequencies (from same 100 random U‐dives mentioned above) was apparent, inferring a distinction between activity/behavior types (Figure S3).

## 4 DISCUSSION

### 4.1 Angular velocity as a metric for animal movement

Movement of the body trunk, nearly always involves some degree of angular rotation *(*cf. López et al., 2016), which is perhaps why rotational indices were suggested as a potential activity index two decades ago (*c.f.* Hochscheid & Wilson, 1999). We were able to show a link between activity extent (as manifest by VeDBA) and angular velocity about all three dimensions of rotation, indicating that movement, in our example animal at least, was rarely confined to a specific axis (Figure 1). However, the rate of change of yaw was much higher than the equivalent in pitch and roll (Figure 7). This is not surprising, not least because full rotation about the pitch and roll axes necessitates body inversion, whereas in yaw it does not. In addition, turning about the yaw axis is an important component of behavior for most animals, particularly during navigation, escape, search, foraging, and hunting strategies (Ballerini et al., 2008; Wilson et al., 2013). For ostensibly technical reasons however, yaw metrics seem to have been largely overlooked in animal activity and behavior studies (excepting studies using gyroscopes, e.g., Kawabata et al. (2014)).

The extent to which yaw angle changes compared to pitch or roll (metrics for angular turn extent are about 4 times higher for yaw than pitch or roll in our turtles—cf. Figure 1) indicates that yaw likely has greater scope and range than pitch or roll as a behavior identifier. This will be particularly the case as the extent of any angular turns decreases so that recorded changes impinge on the resolution capacity of the sensors, as may particularly be the case in very slow‐moving animals. For these reasons, we have primarily focused on the yaw dimension of rotation to demonstrate its utility for assessing both general‐scale differences in activity extent and fine‐scale behavior‐specific movements. Care should be taken to determine that this is the case for the study species though, since animals engage in a variety of rotational movement about their principle axes (cf. Tinbergen, 1963). Indeed, a more sophisticated approach could consider pitch‐, roll‐, and yaw‐specific rotations together as signatures of particular behaviors (cf. Figure 5b).

In a similar manner, consideration of multiple metrics for angular changes in yaw over time is complimentary. For example, periods of high AVeY do not necessarily correspond with high turn extent (cf. Figure 3), but indicate rapid, but nonextensive, changes in heading. This can be symptomatic of a particular behavior such as searching by an animal following environmental cues (cf. Basil & Atema, 1994). Indeed, our study turtles often exhibited activities consisting of high AVeY, signifying quick transitions between small turn extents, with such oscillating yaw movements accompanied by a high degree of directionality likely resulting from benthic surveying (Figure 5a_1_). By contrast, great turn extents in a particular directional plane shows circling behavior (cf. Figure 4a_2_, SI_2_), important for a suite of situations ranging from birds soaring on thermals (Williams et al., 2018) to turtle courtship (Schofield, Katselidis, Pantis, Dimopoulos, & Hays, 2007). Importantly though, the lack of any rotation about the yaw axis can equally be used (in conjunction with other sensor outputs) to define resting behavior more appropriately than can be done using accelerometers alone (Figure 4). This is especially the case in slow‐moving species where yaw metrics are of particular value for much longer‐term investigations (Figure 6). Thus, aggregation of mean epochs of, for example, hourly AVeY estimates in relation to the accumulation of the number of various turn extents, can highlight minimal, but important, activity and provide detailed changes of activity over days. Such a broad approach is particularly relevant for reptiles (cf. Shine, 2005) and many invertebrates (cf. McLeese, 1973) where body orientation changes can occur over much longer time periods than is normal for mammals or birds and for which acceleration data serve well to define activity.

Tri‐axial magnetometers have two main drawbacks though: Firstly, being a vector field sensor, only two rotational degrees of freedom are measured at any one time and so angular rotation cannot be resolved should one sensor axis align with respect to the Earth's magnetic inclination lines (cf. Martín López et al., 2016). Secondly, the derivation procedure of pitch and roll (required in the computation of heading) breaks down during bouts of fast, erratic behavior (i.e., during rapid cornering; cf. McNarry, Wilson, Holton, Griffiths, & Mackintosh, 2017), which can cause subsequent inaccuracies of all three rotational axis derivatives (Noda et al., 2012). Choice of study animal, tag attachment method, animal location on Earth, and the period over which any differential is calculated are key parameters to consider when assessing the likelihood and extent of such limitations.

### 4.2 Relationship between AVeY and VeDBA

VeDBA been used extensively as an acceleration‐based proxy for activity‐specific energy expenditure (cf. Jeanniard‐du‐Dot, Guinet, Arnould, Speakman, & Trites, 2017; Stothart, Elliott, Wood, Hatch, & Speakman, 2016). This is based on the theoretical understanding that movement of most vertebrates is the main factor in modulating energy expenditure (Gleiss et al., 2011). The more vigorous the movement, the more energy is expended in muscular contraction, with any change in measured body acceleration being proportional to the muscular forces that displaced the animal's body (and therefore the attached sensor) (according to Newton's first law) (Miwa et al., 2015). Consequently, the integrated measure of dynamic acceleration from each of the three spatial dimensions has been proposed to reflect the mechanical equivalent to energy expenditure involved in movement.

We documented a clear relationship between VeDBA and AVeY across each turtle (Figure 2a), and this is presumably because turning comprises an appreciable fraction of overall movement costs (McNarry et al., 2017). Specifically, a body that moves in a circular motion (of radius *r*) at constant speed (*v*) is always being accelerated at right angles to the direction of motion (toward the center of the circle of magnitude *v* ^2^/*r*). Given that Force = mass × acceleration (*F* = m × (*v* ^2^/*r*) and it is typically the animal that supplies the force through muscular contraction (Halsey et al., 2011), more acute and higher frequency turns are associated with greater energy expenditure (McNarry et al., 2017) (Figure 2a,b). Taken together then, assessing the frequency and extent of turns can be helpful for understanding aspects of animal energy expenditure and the motivations behind behavior (however identified), since the degree and associated cost of angular rotation is modulated by movement decisions (Vásquez, Ebensperger, & Bozinovic, 2002). It is worth noting however that turn costs increase nonlinearly with speed, and thus, it is far more efficient to turn while stationary/slow‐moving, in order to minimize the perpendicular forces and duration of a turn (cf. McNarry et al., 2017). This may partially explain the spread of VeDBA values during acute turns (Figure 2b), and why AVeY does not necessarily scale nicely with VeDBA over time (Figure 4b_2_, 5c, and 6a).

### 4.3 Advantages of AVeY over acceleration estimates

Acceleration measured by animal‐attached tags is susceptible to the specific site of tag placement, so that data from tags that are deployed on, for example, animal carapaces that vary in morphology (including some; reptiles, crustaceans and gastropod species), are particularly likely to incur this problem, making DBA‐type metrics difficult to compare between individuals (cf. Wilson et al., 2020). The problem is also likely to be manifest to some extent even when animals are morphologically similar, simply because the researchers do not place the tags identically on all individuals. Yaw metrics such as those proposed here (AVeY and angular extent) do not suffer from this problem because they are corrected in their derivation (see Section 2). In fact, the difference in susceptibility to tag orientation between accelerometer‐ and magnetometer‐derived metrics appears within our turtle data, which showed markedly different VeDBA versus AVeY relationships for the different individuals (Figure 2a). We note though that the derivation of pitch and roll becomes problematic when they approach ±90°, when *x*, *y*, and *z* values can become inversed. As such, large offsets in position would be problematic, since it restricts the range for accurate measurements of pitch and roll to be made (consequently affecting the accuracy of yaw). This assumption, therefore, breaks down for animals that change orientations frequently at angles greater than perpendicular from their longitudinal and lateral axes of “normal” posture. However, this is less problematic for slow‐moving animals (such as the turtle).

The broad use of DBA metrics as a measure of movement and energy use for many vertebrates to date indicates their utility. However, for turtles, the narrower operational range of VeDBA increases the contribution of relative error from discrepancies in tag placement, which, in turn, can make separation of energy‐specific behaviors between individuals more challenging. This was the case in our data, with an extremely low range of VeDBA estimates and notable offsets between turtles (Figure 2a). Furthermore, various scales of AVeY reported here revealed repetitive patterns in rotational movement that were not evident in acceleration data during short‐term behavioral bouts (Figures 4a_2_ and 5a_1_). We suggest that, in its simplest form, fine temporal responses of orientation may indicate a change in “state”, presumably from an underlying sensory input or physiological demand and this may not always be reflected from the culmination of dynamic movement as manifest by accelerometers (Figure 5c). Crucially, since angular velocity about the three axes of rotation is not affected by discrepancies of tag placements (though see above), it provides a standardized comparator of movement between individuals.

Changes in the recorded acceleration may not always result from movement arising from the limbs. These may, for example, arise from external forces acting on the animal, such as current vectors in the water (Robert‐Coudert & Wilson, 2004), which will tend to translate the body rather than rotate it. We suggest that AVeY and AAV should be used as a measure for activity that is complimentary to DBA, being particularly valuable when DBA‐type metrics are weak and when behaviors can be exemplified according to the scale (or lack of) and pattern of angular rotation exerted.

Lastly, while this study does not provide specific ground‐truthing of the exact “type” of behavior involved, clear patterns emerge using this approach that would otherwise be left unresolved if only acceleration estimates were considered (Figure 7). The advantages of animal‐attached logging systems include to pry more deeply into the movement ecology of animals independent of direct observation, especially when observer monitoring is difficult, such is the case with free‐ranging turtles. However, inference without ground‐truthing does not determine the finding irrelevant (Collins et al., 2015). Previous studies have made subjective behavioral assignments using prior knowledge and an objective intuition of what acceleration‐based measures infer (cf. Laich, Wilson, Quintana, & Shepard, 2008, Collins et al., 2015). With regard to turtles, underwater behavior has mostly been inferred from speculation based on “dive profiles” (2D representation of depth vs. time). The fact that trends of movement can be resolved more finely using indices of angular velocity, especially during slow scales of movement (pertinent in this study), only aids in objectively quantifying behavioral differences. We suggest that variation of heading in a specified time frame is intrinsically linked to the functional behavior motivation exhibited. Estimates that are consistent in the magnitude and pattern of change reveal a behavior that is consistent in its vector of imparted motion (or rotation) (cf. Figure 5, Figure S3). Crucially however, even “noisy” variations in AVeY may indicate more notable differences between activity‐specific behaviors that acceleration estimates do not (Figure 7).

## 5 SUMMARY

This work assessed the value of animal heading (body orientation) and associated metrics for quantifying animal activity and for helping define behaviors. Our results show that incorporating the frequency and extent of yaw axis rotations at various temporal scales aids in resolving patterns of movement beyond that which acceleration‐based metrics alone can detail. We suggest that yaw‐based metrics should help identify animal behavior and as indices of general activity over both short‐ and long‐term periods, especially (within an energetic context) for slow‐moving animals. It would be useful to have AVeY and AAV metrics compared to oxygen consumption to check their validity as a potential proxy of energy expenditure. Further work should assess the value of AVeY on a suite of terrestrial, aquatic, and volant animals, varying in activity extent and behavior. Judicious choice of sampling intervals and smoothing windows will be important considerations in this.

## CONFLICT OF INTEREST

The authors declare no conflict of interest.

## AUTHOR CONTRIBUTION

**Richard Michael Gunner:** Conceptualization (lead); Data curation (lead); Formal analysis (lead); Investigation (equal); Methodology (equal); Resources (supporting); Software (supporting); Validation (lead); Visualization (lead); Writing‐original draft (lead); Writing‐review & editing (equal). **Rory Wilson:** Conceptualization (equal); Data curation (supporting); Investigation (supporting); Methodology (supporting); Project administration (equal); Resources (supporting); Software (supporting); Supervision (lead); Validation (supporting); Visualization (supporting); Writing‐original draft (supporting); Writing‐review & editing (equal). **Mark Holton:** Methodology (supporting); Resources (supporting); Software (supporting); Supervision (supporting); Visualization (supporting); Writing‐original draft (supporting); Writing‐review & editing (supporting). **Rebecca Scott:** Data curation (equal); Funding acquisition (lead); Investigation (supporting); Methodology (supporting); Project administration (supporting); Resources (supporting); Writing‐original draft (supporting); Writing‐review & editing (supporting). **Phillip Hopkins:** Methodology (supporting); Resources (supporting); Writing‐original draft (supporting); Writing‐review & editing (supporting). **Carlos Duarte:** Funding acquisition (supporting); Investigation (supporting); Project administration (supporting); Resources (supporting); Supervision (supporting); Writing‐original draft (supporting); Writing‐review & editing (supporting).

## Supporting information

Figures S1‐S3

## Data Availability

A large 1 Hz data frame, including tri‐axial accelerometery, tri‐axial magnetometry, pressure, and angular velocity metrics, during bottom phase U‐dives from all turtles has been uploaded to the online open access repository; Figshare (https://doi.org/10.6084/m9.figshare.9862271). The DOI will become active if the manuscript is accepted for publication.
