# Supplementary material for: A new direction for differentiating animal activity based on measuring angular velocity about the yaw axis
Source: Ecol Evol. 2020 Jul 6;10(14):7872–86. doi: 10.1002/ece3.6515 (PMC7391348; doi:10.1002/ece3.6515)
Supplement: Supplementary file 1 — Figures S1‐S3 [file ECE3-10-7872-s001.docx]

**Supplementary Information: Figures 1:3**


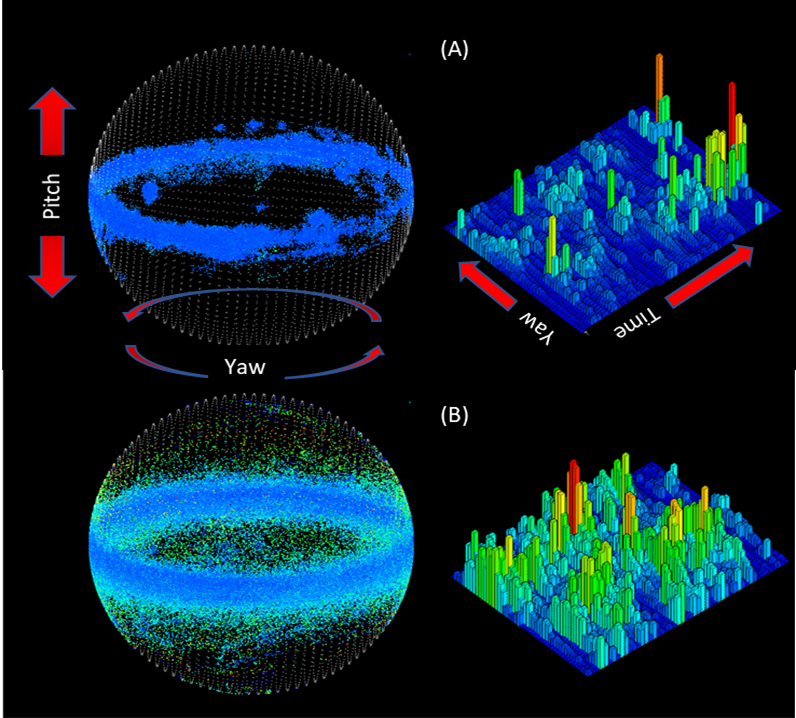


Figure 1. O-sphere visualisation showing the simultaneous distribution of yaw angles (longitude plane and pitch angles (latitude plane) from data during bottom phase of type 1a dives when AVeY remained less than 3 ^o^/s (A) and greater than 3 ^o^/s (B) (coloured according to the magnitude of VeDBA gradient (low: blue –> green 🡪 red: high) (Left). 2D frequency histograms of the heading (0 - 360^o^) over time (bins = 50 for both x and y scales, size and colour intensity of each ‘facet’ represents a greater relative frequency (Right).

**
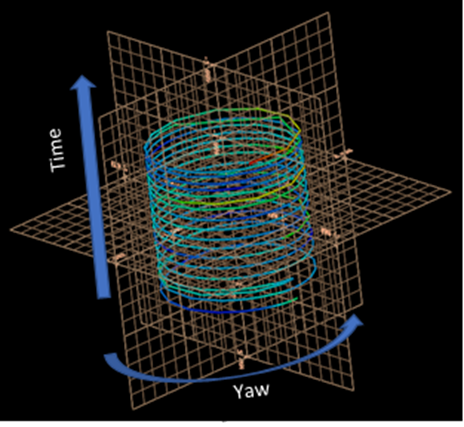
**

Figure 2. Yaw plotted in 3-dimensional space. Time is represented by distance from the base (colour coded according to the Magnitude of VeDBA. Data taken from a bout of circling behaviour (17 revolutions in 3 minutes 45 seconds.


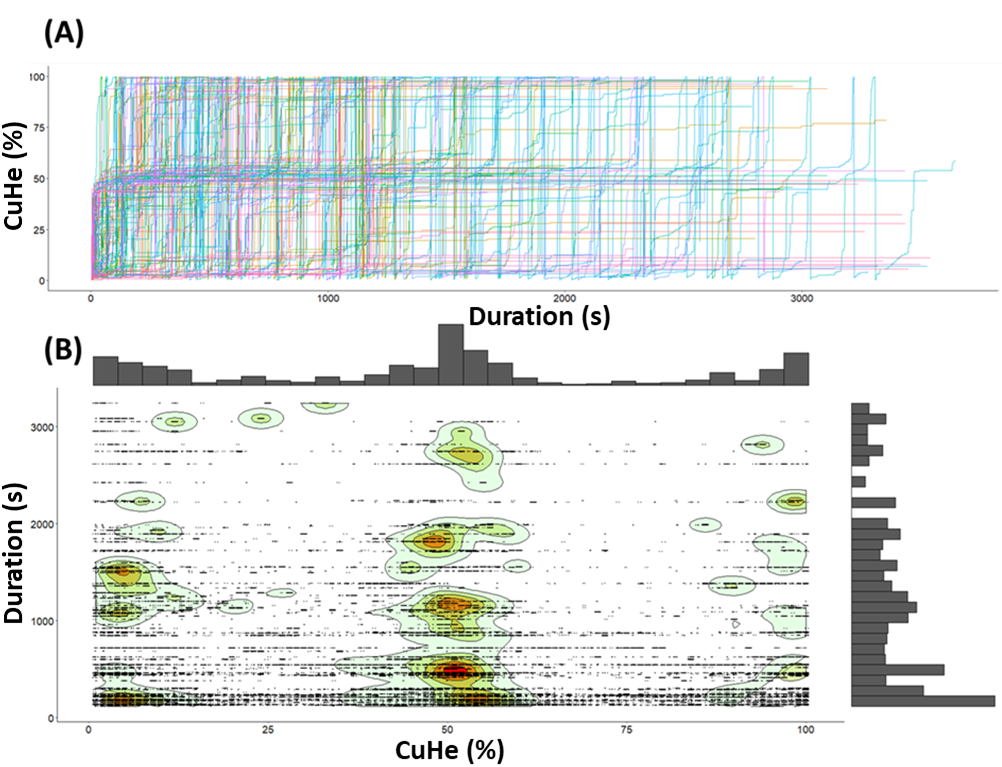


Figure 3. Patterns of CuHe across 100 random U-dives (bottom phase). This is expressed both as a line graph (coloured according to dive number) (A) and a 2d contour scatterplot with marginal histograms illustrating the relationship between the most frequented CuHe percentage and the uninterrupted duration such values were frequented for (before an increment). Note, each time CuHe resets to 0, all angles of the animal’s body circumference have been covered. 1 % increase in CuHe corresponds to the body arc covering an extra 3.6 degrees (not previously covered since the last reset).
